# Perinatal Outcome After Diagnosis of Oligohydramnious at Term

**DOI:** 10.5812/ircmj.11772

**Published:** 2014-05-05

**Authors:** Kolsoum Rezaie Kahkhaie, Fateme Keikha, Khadije Rezaie Keikhaie, Abdolghani Abdollahimohammad, Shahrbanoo Salehin

**Affiliations:** 1 Zabol Medicinal Plants Research Center, Zabol University of Medical Sciences (ZBUMS), Zabol, IR Iran; 2 Department of Obstetrics and Gynecology, Tehran University of Medical Sciences, Tehran, IR Iran; 3 Department of Perinatalogy, Zabol University of Medical Sciences (ZBUMS), Zabol, IR Iran

**Keywords:** Amniotic Fluid, Apgar Score, Birth Weight

## Abstract

**Background:**

Oligohydramnious is threatening condition to fetal health for which some treatment are available and some are under evaluation. Oligohydramnious, is associated with increased pregnancy complication, congenital anomalies and perinatal mortality. There is an inverse relationship between the amniotic fluid index (AFI) and the adverse perinatal outcome. Oligo hydramnlious is clinical condition characteries by amniotic fluid index (AFI) of 5cm or less. Its incidence is 3-5 % of all the pregnancies an accurate and reproducible method of determining abnormality in amniotic fluid volume (AFI) is sonographic asessment of amniotic fluid index (AFI). It often increase the risk of small for gestational age (SGA) and also the incidence of cesarean section, meconium stained, low apgar score and Neonatal intensive care (NICU) admission.

**Objectives:**

The aim of study was to analyze the fetal out come in low risk pregnant women with oligohydramnious at term. This is a prospective, descriptive study.

**Materials and Methods:**

The study was conducted at Amiralmomenin hospital in Zabol for a period of 8 months from 2012/Mar/27 to 2012/Nov/5.It included 100 pregnant women diagnosed with the AFI of or less than 5cm at term. Control group included 300 pregnant women with AFI more than 8cm. Comparison was done between the study group and the control group. Regarding the fetal and pregnancy outcome using chi square and p value, detail were recorded in terms of fetal weight, apgar score at 1 and 5 minutes.

**Results:**

Mode of delivery, NICU admission neonatal death and induction of labour. Oligohydramnious is associated with a high rate of pregnancy complication and increased preinatal morbidity and mortality. Women with oligohydramnious usually have low birth babies.

**Conclusions:**

However, it can expect a safe and good outcome for which proper fetal surveillance and regular antenatal care visits are required.

## 1. Background

Amniotic Fluid (AF) is an important part of pregnancy sac and helps fetal development. Amniotic Fluid has a number of prominent functions like protects the fetus from trauma, maintains body temperature and development of musculoskeletal system by permitting fetal movements growth and development of intestinal tract by swallowing amniotic fluid and it provides essential nutrients to fetus (1). There is a gradual increase in volume with advancing gestation until approximately 31-33 week followed by a significant decrease toward and beyond the estimated date of confinement.

At term (37 and beyond) the average is approximately 750ml, but volume decrease rapidly after 40 weeks (2). Oligohydramnious was defined as an amniotic fluid index (AFI) ≤ 5 cm (3, 4). In 1990, Moore and Cayle defined oligohydramnious as an AFI below the 5th centile for the gestational age (5). Its incidence is 2.3 % of all the pregnancies (6). In 2005 Leeman et al. reported oligohydramnious occurred in about 1 % to 5 % of pregnancies at term (7).

Assessment of amniotic fluid volume by ultra sonography is more reliable (8). It is calculated as the sum of the deepest vertical dimension in each quadrant of the uterus (2, 9). Oligohydramnious is associated with increased pregnancy complication, congenital anomalies and perinatal mortality (6) and it may be associated with uteroplacental insufficiency, idiopathic fetal growth restriction (IUGR), premature rupture of the fetal membranous, fetal hypoxia, meconium stained fluid and or post maturity syndrome (10). Oligo hydramnious can also be an idiopathic finding in a woman who had low risk pregnancies and no medical or fetal complication (7). Sequel of oligohydramnious can be fetal demise, pulmonary hypoplasia, facial and skeletal deformities. Reduced amniotic fluid may predispose to umbilical cord occlusion and increase the risk of fetal hypoxemia and will affect the Apgar score of baby at birth (11).

It has been observed that antepartum or intra partum AFI ≤ 5 cm is associated with a significant increase in risk of lower segment caesarean section for fetal distress and low apgar score at 5 minute(apgar score < 5) (12). Patients with AFI ≤ 5 cm should be admitted to the hospital (13).

Determination of the optimal time of delivery is necessary and labor should not be prolonged (14). Oligohydramnious is the late sign of malnutrition. In adequate nutrition is the second important cause of IUGR and associated complication (15). Current local practices rely heavily on AFI estimation, particularly in the management of prolonged pregnancy and IUGR (16-18). The role of AFI as an isolated predictor on the fetal outcome needs to be checked not only in prolonged pregnancies, but also in other frequency managed high-risk pregnancies.

## 2. Objectives

The purpose of this study was to assess low amniotic as a predictor of perinatal outcome in low risk pregnancies at term.

## 3. Materials and Methods

This case-control prospective study was conducted at Amiralmomenin hospital in Zabol for a period of 8 months from 27 Mar 2012 to 5 Nov 2012. Pregnant women were divided to two groups. 100 consecutive pregnant women with AFI ≤ 5cm with low risk pregnancies at term were included in group A and 300 women with AFI ≥ 5cm and ≤ 20cm were included in group B. Inclusion criteria were women with singleton, term, non anomalus pregnancies with intact membrane evidence of IUGR, previous cesarean section, post term pregnancies, previous perinatal loss, recurrent missed abortion, medical disorder like Dm, hypertention and cardiac disease were excluded from this study. Both groups were matched for age, parity, gestational age and intact membranes. All women were followed up until delivery and pregnancy and perinatal outcome were recorded using chi square (x2), and p value was calculated to determine the statistical significance.

## 4. Result

During the study period, there were 100 Patient with AFI ≤ 5cm and 300 Patient with AFI > 5cm.Maximum numbers of women were in the age group 25-35 year (46.9 %). 49 % women in oligohydramnios group were primigravida. Cesarean section was done in 20.2 % in group A and in control group was 8.6 % (Table 1). 

**Table 1.** Pregnancy and Perinatal Outcome

	Oligohydramnious Group, No. (%)	Control Group, No. (%)
**Caesarean section**	20 (20.2)	26 (8.6)
**Distress**	9 (8.9)	7 (2.4)
**Birth weightless than 2.5 kg**	29 (29)	53 (17.6)
**Induction**	42 (42)	38 (12.6)
**APGar scoreless than 7at 5minutes**	8 (4.7)	14 (4.7)
**Use by**	1 (1)	3 (1)
**Admitted to the NICU**	1 (1)	0 (0)

There was a significant difference in Cesarean section rate between two groups. So that the odd’s ratio in Oligohydramnious groups was 2.79 greater than control groups, and also statistically difference in cause of cesarean section in two groups so that the fetal distress in Oligohydramnious groups 4.01times greater than control groups. There was an increased incidence of SGA among women with Oligohydramnious and control groups so that in Oligohydramnious SGA 1.91 % was greater than control groups. There was significant statistically difference in induction of labour so that the induction of labour in Oligohydramnious was 3.22 times greater than control groups and There was no difference in incidence of instrument delivery APGar score < 7 at 0 and 5 minute between two groups and also no difference found throughout the length to stage in NICU.

## 5. Discussion

Estimation of amniotic fluid volume is an integral part of antenatal surveillance (19). Reduce amniotic fluid carries an increased risk of an intrapartum complication in high-risk pregnancies (6, 20). Relationship between sonography detected Oligohydramnious and perinatal morbidity, and mortality has been well established by manning and platt (21). Garmel et al. supported that 67 % of women with Oligohydramnious were nulliparous (22) and Charu etal supported that 66 % of women were nulliparous (23). While we observed that 49 % of women to be primigravida. Chauhan et al. (12) concluded that AFI < 5cm is associated with increased risk of Caesarean section for fetal distress and low apgar Score at 5 minute, in this study 20.2 percent of women were delivered by Caesarean section.

Results of Umber A (24) and Jandial et al. (23) which showed significantly increased incidence of Caesarean section and non-reassuring fetal heart rate in women with low AFI. In this study 20.2 %percent of women were delivered by caesarean section. Saro et al. (25) noted a significantly higher rate of fetal distress and low apgar score in women with AFI 5cm. Golan et al. (26) reported a low apgar score at 5minute in 4.6 babies in contrast to a figure of 9.9 %noted by us. Casy et al. (6) reported 6.4 % perinatal death while we conducted in 5.9 % perinatal death. Meconium staining is an indicator of fetal distress and has its own complication in the newborn. In this study, there was no significant difference in the incidence of meconium in two groups. This results were consistent with certain studies (24). To conclude Oligohydramnious is associated with high rate of pregnancy complication and increase perinatal morbidity and mortality. We believe that AFI assessed ante partum, and intrapartum would help to identify women who need increase ante partum surveillance for pregnancy complication. Women with oligo hydramnios usually has low birth babies but can expect the safe and good outcome for which proper fetal surveillance and regular antenatal care visits are required.
